# The Role of Wnt and R-spondin in the Stomach During Health and Disease

**DOI:** 10.3390/biomedicines7020044

**Published:** 2019-06-19

**Authors:** Anne-Sophie Fischer, Michael Sigal

**Affiliations:** 1 Department of Hepatology and Gastroenterology, Charité University Medicine, 10117 Berlin, Germany; anne-sophie.fischer@charite.de; 2 Department of Molecular Biology, Max Planck Institute for Infection Biology, 10117 Berlin, Germany; 3 Berlin Institute of Health, 10117 Berlin, Germany

**Keywords:** Wnt signaling, gastric cancer, R-spondin, Helicobacter pylori, stem cells, Lgr5+ stem cells, Axin2

## Abstract

The Wnt signaling pathway is one of the most prominent developmental signals. In addition to its functions in development, there is emerging evidence that it is also crucial for various organ functions in adult organisms, where Wnt signaling controls tissue stem cell behavior, proliferation and differentiation. Deregulation of Wnt signaling is involved in various pathological conditions and has been linked to malignant tissue transformation in different organ systems. The study of the Wnt signaling pathway has revealed a complex regulatory network that tightly balances the quality and strength of Wnt signaling in tissues. In this context, R-spondins are secreted proteins that stabilize Wnt receptors and enhance Wnt signaling. In this review we focus on new insights into the regulatory function of Wnt and R-spondin signaling in the stomach. In addition to its function in the healthy state, we highlight the connection between Wnt signaling and infection with *Helicobacter pylori (H. pylori)*, a pathogen that colonizes the stomach and is the main risk factor for gastric cancer. In addition to experimental data that link Wnt signaling to carcinogenesis, we discuss that Wnt signaling is affected in a substantial proportion of patients with gastric cancer, and provide examples for potential clinical implications for altered Wnt signaling in gastric cancer.

## 1. Introduction

Wnt, originally named as int1, was discovered in 1982 when Roel Nusse and Harold Varmus performed research on oncogenic retroviruses [1]. However, a few years later, in 1987, it became apparent that int1 is a homologue to the gene Wingless, which was discovered in 1980 by Christiane Nüsslein-Volhard and Eric Wieschaus [2,3,4]. Subsequently, Wnt signaling has been recognized as one of the major developmental signals, which is highly conserved and present in all multicellular organisms and known to be important for cellular diversity during development. 

While it is crucial for development, more recently the role of Wnt signaling in adult tissues has become a major focus of research. In this context, it emerged as a critical pathway that regulates stemness, and self-renewal and cellular differentiation in several tissues [5]. In addition, Wnt signaling has been recognized as one of the most prominent driver pathways in cancer. Mutations that lead to uncontrolled activation of Wnt signaling are found in many cancers originating in the gastrointestinal tract. Around 93% of patients with colorectal cancer have mutations in the Wnt signaling pathway [6]. Although the mutational sequence from normal tissue to cancer is less clear for gastric cancer, aberrant Wnt signaling has been shown to play a significant role for gastric carcinogenesis, and Wnt signaling mutations are found in a substantial proportion of patients with gastric cancer. Here, we focus on the role of Wnt signaling in healthy gastric tissue as well as in the context of carcinogenesis. Furthermore, we highlight some recent studies that indicate an association between aberrant Wnt signaling and the prognosis for gastric cancer patients, as well as its role as a target for therapeutic intervention.

## 2. Wnt and R-spondin Signaling

In the healthy stomach, Wnt signaling is induced by an interaction of secreted lipid-modified glycoproteins that act as ligands for Wnt receptors. The lipid modification, so-called palmitoylation, is thought to be important for the short range signaling of Wnt ligands, allowing spatial diversity within the tissues [7]. There are 19 different Wnt ligands present in humans and mice [7]. The variety of Wnt ligands and receptors leads to the induction of various cellular signaling pathways, that overall can be divided into canonical and non-canonical pathways. While the canonical pathway involves beta-catenin translocation into the nucleus, the non-canonical pathway is beta-catenin independent and involves the planar cell polarity pathway as well as the non-canonical Wnt/calcium pathway. Whether Wnt signaling is transmitted through the canonical or the non-canonical pathway depends on the type of Wnt ligand on one hand and on the set of receptors of the cell on the other [8]. Wnt3A, for example, is classically thought to induce canonical Wnt signaling but has also been shown to act through the non-canonical signaling cascade [9]. In contrast, Wnt5A has been reported to bind to several receptors and induce both Wnt signaling pathways [10].

**Figure 1:**
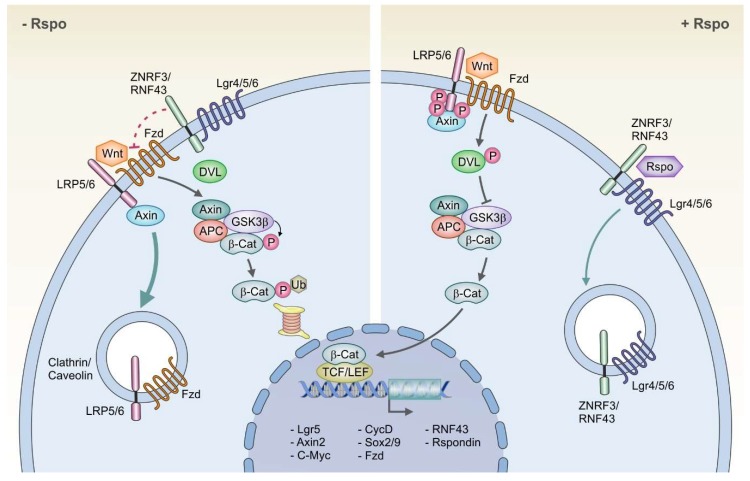
The Wnt and R-spondin signaling pathway: In the absence of R-spondin (-Rspo), the membrane ubiquitinase zinc and ring finger 3/ ring finger 43 (ZNRF3/RNF43) associates with the Wnt receptor complex Frizzled/ Low-density lipoprotein receptor-related protein 5/6 (Fzd/LRP5/6), inducing its internalization and preventing its Wnt dependent phosphorylation, thereby inhibiting the downstream Wnt signaling cascade. In the presence of R-spondin (+Rspo), the ubiquitinase ZNRF3/RNF43 is internalized, enabling Wnt dependent phosphorylation of LRP5/6 and Dishevelled (DVL) as well as inhibition of glycogen synthase kinase-3 beta (GSK3beta). This then leads to the stabilization of the transcription factor beta-catenin (β-cat), its translocation in the nucleus and the subsequent expression of Wnt target genes.

In the gastrointestinal tract, the canonical pathway has been extensively studied and has been shown to regulate cell fate and proliferation [8]. It has also been linked to stem cell dynamics, while its deregulation leads to cancer initiation and progression [11,12,13,14,15]. Canonical Wnt signaling is transmitted through the interplay of the 7-transmembrane receptor Frizzled and the single membrane receptor Low-density lipoprotein receptor-related protein 5/6 (LRP5/6). This interplay induces a conformational change of the receptors with subsequent phosphorylation. The phosphorylation then enables the receptor complex to inhibit glycogen synthase kinase-3 beta (GSK3beta) [16]. GSK3beta is part of a destruction complex that also includes Axin and Adenomatous Polyposis Coli (APC) and that in the absence of the Wnt receptor activation leads to the destabilization of the transcription factor beta-catenin. However, upon inhibition of GSK3beta, beta-catenin is protected from degradation and is translocated to the nucleus, where it interacts with the transcription factors Tcf/Lef and subsequently results in the expression of Wnt target genes [17] (see Figure 1). These include genes that are essential for proliferation, self-renewal, metabolism, and epithelial-mesenchymal transition [8].

The Wnt-receptor LRP5/6 is predominantly expressed in epithelia that are highly proliferative and in which cells that derive from dividing stem cells are constantly pushed along the epithelial unit, such as gland or crypt. Importantly, those epithelia are dependent on sufficient Wnt signaling for maintaining healthy homeostasis [18]. 

Most of the research on Wnt signaling in the gastrointestinal tract has been done in the intestine and colon. This is probably due to the fact that mutations in Wnt signaling are found in almost all patients with colorectal cancer [6]. The role of Wnt signaling in the stomach is less clear; however, recent data have shown that it is a relevant pathway in the adult stomach. Various Wnt ligands and receptors, as well as molecules that can modify Wnt signaling, are expressed in the mouse stomach. Moreover, gastric stem cells express classic Wnt target genes such as Axin2 and Leucine-rich repeat-containing G-protein coupled receptor 5 (Lgr5) [15,19].

### Potentiation of Wnt Signaling by R-spondin

As Wnt signaling has a large influence on the overall epithelial architecture, the tight regulation of Wnt activity within the tissue is essential for its integrity. While Wnt ligands are present throughout the stomach tissue, Wnt signaling, reflected by the expression of target genes, is limited to the base of the glands in the antrum [15].

To clarify this discrepancy, R-spondin3 was identified as a critical stromal niche factor, produced by myofibroblasts of the lamina muscularis mucosae, specifically in the vicinity of the gland base [15]. 

Four different R-spondin homologues have been described. All of them are secreted proteins, produced in the endoplasmic reticulum, and each of them can bind to all three Lgr5 homologues (Lgr4, Lgr5 and Lgr6) [20]. Nevertheless, they are present in different types of tissues and also carry out different functions during development and in the adult organism [21].

In the absence of R-spondin, activation of the Wnt signaling pathway results in activation of target genes, including the transmembrane E3 ubiquitin ligases ring finger 43 (RNF43) and zinc and ring finger 3 (ZNRF3), which are functional homologues [20,22]. Upon their integration in the cell membrane, they multiubiquitinate the Wnt receptor complex Frizzled/LRP5/6, leading to its internalization and lysosomal degradation [22,23]. This negative feedback loop functionally limits Wnt signaling. Accordingly, a simultaneous knockout of RNF43 and ZNRF3 in the mouse intestine resulted in strong proliferation of the stem cell compartment [23]. Nevertheless, loss-of-function mutations of RNF43/ZNRF3 only lead to enhanced Wnt susceptibility of the respective cell, and therefore, the presence of Wnt is still necessary to induce hyperproliferation. This is in contrast to the loss-of-function mutations of Wnt inhibitors that are further downstream in the Wnt signaling pathway, such as APC, whose downregulation or knockout can autonomously promote cell proliferation [22,23].

R-spondin binds to the extracellular domain of RNF43/ZNRF3 and to Lgr4/5/6, leading to their physical association and internalization into the cytoplasm [22,23]. This loss of the inhibitory ubiquitinase RNF43/ZNRF3 from the membrane thus stabilizes the Wnt receptors, enabling their activation and thereby the potentiation of Wnt signaling [22,24]. In the stomach, R-spondin3 is a critical molecule that controls Wnt signaling and induces proliferation of Axin2+/Lgr5− stem cells [15]. The lack of Lgr5 receptors in this cell population is likely compensated by the presence of its homologue Lgr4, which can also serve as a receptor for R-spondin and is expressed in a broad population of gastric epithelial cells, including Axin2+/Lgr5− stem cells [15]. R-spondin thereby significantly contributes to stem cell and gland turnover dynamics [15]. Accordingly, adenovirus-induced overexpression of RNF43 in gastric cancer cells reduces Wnt signaling and leads to a significant decrease in stem cell properties and tumorigenicity [25], further pointing towards a significant involvement of R-spondin in gastric carcinogenesis. 

While R-spondin molecules potentiate Wnt signaling, the functional impact of Wnt ligands interacting with the Fzd/LRP5/6 receptor and of R-spondin interacting with Lgr5 has been shown to be synergistic but non-redundant in the context of small intestinal stem cells [26]. Inhibition of R-spondin signaling through expression of soluble Lgr5 and ZNRF3 ectodomains by adenoviral infection led to a loss of Lgr5+ stem cells in vivo, and this loss could not be rescued through an overstimulation of Wnt signaling via Fzd/LRP5/6 receptor [26]. The authors therefore concluded that only the amplitude of R-spondin, but not of Wnt, defines the number of Lgr5+ stem cells in the intestine, whereas Wnt ligand interaction with Fzd/LRP5/6 is rather important for Lgr5-driven epithelial turnover [26]. 

## 3. Wnt and R-spondin Signaling and Gastric Gland Homeostasis

The stomach is anatomically divided into two main parts: the stomach body or corpus, which contains acid producing parietal cells, and the distal part, or antrum.

**Figure 2:**
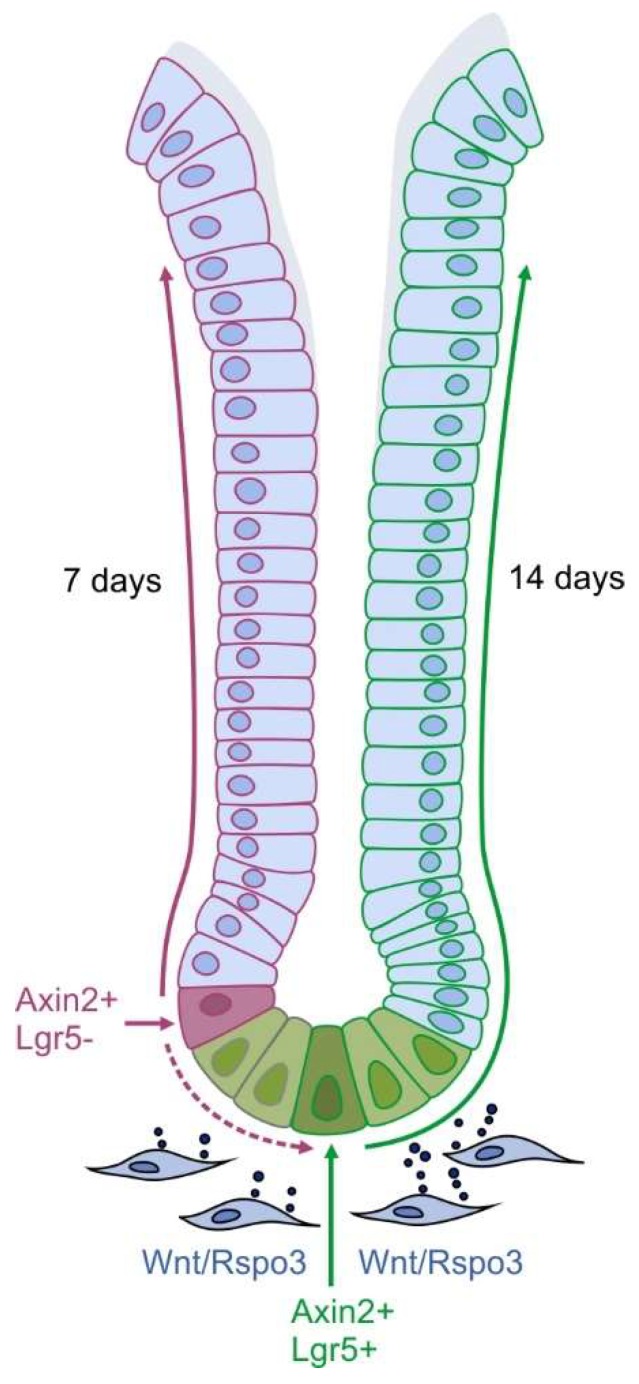
Composition of the homeostatic antrum gland: The stem cell compartment is located at the base of the gland consisting of a basal Axin2+/Lgr5+ stem cell population and a further apical Axin2+/Lgr5- stem cell population. Myofibroblasts of the lamina muscularis mucosae produce R-spondin3 and Wnt, which fuel the proliferation of both stem cell populations. Their progenies are pushed towards the top of the gland, thereby renewing the antrum gland within 7 days by Axin2+/Lgr5- cells or within 14 days by Axin2+/Lgr5+ cells.

The antral epithelium is highly proliferative and shows a rapid migration of differentiating cells, which are constantly shed into the lumen, resulting in a full gland turnover within one to two weeks [19,27]. Antrum glands have a characteristic organization: the base contains Axin2+/Lgr5+ cells, which are both Wnt target genes, and these cells have been shown to repopulate entire antrum glands [19]. Right above this compartment, the more proliferative Axin2+/Lgr5− cells are present, that are most likely the main drivers of tissue regeneration under homeostatic conditions [15]. Furthermore, upon Lgr5+ cell depletion, Axin2+ stem cells can repopulate the entire gastric gland and thereby might play an essential role in the compensation of tissue injury [15]. Both stem cell populations give rise to progenitor cells as well as to differentiated cell types such as gastrin-producing enteroendocrine cells, Muc6+ gland base mucous cells and Muc5AC+ mucous pit cells, as well as Tuft cells [15,19]. The constant proliferation of the stem cells pushes their progenies further up the gland, where they differentiate during their migration and are finally shed into the lumen (see Figure 2). 

Since both Axin2+/Lgr5+ as well as Axin2+/Lgr5− cells are capable of repopulating the gland, the stem cell compartment seems to contain different non-homogenous populations of cells. In fact, the Lgr5+ stem cells have a rather slow turnover time of 10–14 days and a low proliferation rate of 10% to 20% [28]. In contrast, Axin2+/Lgr5− stem cells repopulate glands within 7 days [15].

The heterogeneity of cells in the gland base that are able act as stem cells demonstrates the cellular plasticity in this compartment, and it is likely that interconversion between the two cell types occurs. Of note, as both Lgr5 and Axin2 are Wnt target genes, Wnt signaling could be crucial for the stem cell identity, irrespectively of the proliferative state of the cell. It is possible that different subpopulations of gastric antrum stem cells are differentially regulated, allowing activation or deactivation of a particular population based on the environmental context, which may be particularly important for a rapid response to epithelial damage and an efficient repair of injured gastric epithelium. Accordingly, R-spondin3 leads to an expansion and increased proliferation of Axin2+/Lgr5− cells, whereas Axin2+/Lgr5+ cell proliferation is rather silenced. 

Since the expression of Rspo3 increases in the context of injury (see below), Lgr5+ cell silencing may represent a strategy to protect this cell population in the context of injury. In this context, further investigations may shed light onto which other signals are involved in fine-tuning the gland base and regulating the differential behavior of different stem cell subpopulations, which finally determines the architecture of the gland.

In the corpus, the gland organization differs from the one in the antrum, which is particularly reflected by the presence of acid-producing parietal cells throughout the corpus gland, as well as by secretory chief cells in the base of the glands. Although Lgr5+ cells were identified throughout the gastrointestinal tract, they were not detected in the corpus in the original studies that used Lgr5− EGFP-ires-CreERT2 mice to follow the fate of Lgr5-expressing cells [19,29]. However, this was likely due to technical problems of the mouse line rather than a reflection of the biological state of the gland [11]. Eventually, it has been shown that the expression levels of Lgr5 are similar in the corpus and in the intestine, and that Lgr5+ cells are found in the base of corpus glands in the chief cell compartment [30].

In the context of Wnt signaling, an alternative stem cell marker used was Troy, which is also a Wnt target gene and is furthermore enriched in Lgr5+ intestinal stem cells [31]. Using this marker, as well as Mist1, another marker of chief cells, Stange et al. have demonstrated that a particular subpopulation of gland base chief cells does express Wnt target genes, including Lgr5, Axin2 and RNF43/ZNRF3 [30]. These cells did not proliferate under homeostatic conditions, but after applying the chemotherapeutic antiproliferative drug 5-FU in vivo, Troy+ cells increased their proliferation rate and repopulated the injured glands. Consequently, Troy+ cells and the Wnt signaling pathway are considered as particularly responsible for restoring tissue integrity after injury of the actively proliferating stem cell compartment [30]. 

More recently, the availability of a new reporter for Lgr5 has confirmed that, under homeostatic conditions, corpus Lgr5+ cells only rarely contribute to the gland homeostasis [11]. In contrast, Mist1+ Lgr5− cells in the isthmus were shown to be more proliferative and to more efficiently regenerate the corpus gland [32]. While this may implicate that Lgr5+ cells in the base are dispensable for homeostasis, Leushacke et al. showed that depletion of Lgr5+ cells using Lgr5-DTR mice does have a profound effect on the corpus gland architecture. They also described that upon depletion of Lgr5 cells, remaining Troy+ cells may fuel the regeneration, which suggests a co-existence of multiple Wnt-responsive cells with stem cell properties in the corpus [11]. 

Nonetheless, it remains unclear how the functional switch of gland base chief cells to proliferative stem cells occurs upon injury. Leushacke et al. characterized the expression pattern in Lgr5+ cells upon injury and found an upregulation of Wnt related genes such as Mmp7, which is a direct target gene of beta catenin/TCF4, and Sostdc1, which is a Wnt inhibitor [11]. These results point towards a potential role of Wnt signaling in regulating the cellular state in the corpus gland base upon injury, although the full mechanism remains unclear. 

## 4. From Healthy Tissue to Cancer: Link Between Damage, Wnt Signaling and Cancer

The gram-negative bacterium *Helicobacter pylori (H.pylori)* is a WHO class I carcinogen and the main risk factor for gastric cancer [33]. *H. pylori* is able to colonize gastric glands, and once colonization is established the bacterium has evolved to persist for decades [34]. However, only a small fraction of patients with *H. pylori* infection will develop gastric cancer [34,35]. 

There are several host-related factors as well as bacterial virulence factors that are linked to an increased risk for pathology [36]: CagA is the most prominent virulence factor of *H. pylori*. Upon adherence to epithelial cells, *H. pylori* uses its type four secretion system, which acts as a molecular syringe, to inject CagA into host cells. Upon translocation, CagA is phosphorylated and interferes with signal transduction within the host cells. In the context of Wnt signaling, it has been shown that CagA can interfere with GSK3beta-induced degradation of beta-catenin and thereby lead to the stabilization of beta-catenin, which is then translocated to the nucleus and initiates the expression of Wnt target genes [37]. This is further supported by a study that shows that *H. pylori* positive gastric cancer samples have a significantly higher beta-catenin expression than those of *H. pylori* negative cancer tissues [38]. Furthermore, CagA has been linked to epithelial-mesenchymal transition by depleting GSK3beta [39]. Yet other papers demonstrated that CagA positive *H. pylori* induces upregulation of stem cell associated markers such as Axin2 [40], Nanog and Oct4 [41] and thereby potentiates epithelial cell proliferation [40]. 

Apart from direct effects of CagA and *H. pylori* on Wnt signaling, infection also interferes with Wnt signaling on the tissue level through intercellular communication. As pointed out above, Wnt signaling in the stomach is not a cell intrinsic feature of the cells but is instead largely controlled and induced by the microenvironment. In this context, infection with *H. pylori* has been shown to interfere with the homeostatic division of stem cells within the antral gland, resulting in an increased number and division rate of Axin2+ cells [15]. This is substantially driven by stromal cells surrounding the gland, which secrete R-spondin3. This factor is indeed present at increased levels upon infection, driving an expansion of Axin2+ stem cells [15]. In contrast, mice that lack R-spondin3 specifically in Myh11+ myofibroblasts have a significant reduction of epithelial Wnt target gene expression and do not show an expansion of stem cells upon infection [15]. Of note, stem cell responses to infection are not triggered by infection per se, but are mainly driven by a subpopulation of *H. pylori* that are able to invade the gland and colonize the apical junctions of the stem cell and progenitor cell pool [27]. This indicates that responses to infection are triggered by an interaction of stem cells with bacteria, while bacteria that interact with the more differentiated cells or are free-swimming do not trigger these responses. Accordingly, it has been demonstrated using primary organoid technology that epithelial immune responses to *H. pylori* are more pronounced when cells are grown in media with Wnt and R-spondin3, whereas the response of differentiated cells grown without Wnt is diminished [42,43]. While the data point towards a link between inflammation and R-spondin signaling, the regulation of R-spondin expression remains not fully understood. Moreover, the consequences of stem cell activation through *H. pylori* infection need to be investigated in more detail. 

## 5. Wnt Signaling in Gastric Cancer

New studies reveal that not only in the colon but also in the stomach the activation of Wnt signaling could represent a critical step in the carcinogenic cascade. Thus, pathologic activation or mutation of the Wnt signaling cascade has been found in around 30% of gastric cancer tissues [44]. Various mechanisms underlying the enhancement of Wnt signaling have been found, including gain-of-function and loss-of-function mutations and epigenetic alterations, as well as changes induced by phosphorylation and miRNA activity [13] (see Table 1).

Within the group of upregulated molecules, different Wnt molecules are enhanced in gastric cancer: For example, Wnt5A has been shown to be significantly upregulated [48], and histological analyses revealed that this upregulation occurs in 30% of gastric carcinomas [49]. Apart from Wnt5A, also other Wnts such as Wnt1 [45], Wnt2B [47], Wnt6 [50] and Wnt10A [51] have been found to be enhanced in gastric cancer tissue [13]. Furthermore, significant upregulation of mRNA for beta-catenin has been reported [52]. 

Apart from that, loss-of-function mutations of genes that inhibit or limit Wnt signaling were observed: Mutations in the APC gene have been reported in 7% [12], 15–18% [53,54] or even 30–34% [52,55] of gastric cancer samples. Inactivating mutations have additionally been found in Wnt inhibitory genes coding for Axin 1 and Axin 2 [56]. Whole genome sequencing also revealed that the ubiquitinase RNF43 is frequently mutated and subsequently downregulated in gastric cancer [25]. In hypermutated gastric carcinomas, 33% of tumors carried a RNF43 mutation [12]. Loss-of-function mutations of the Wnt signaling inhibitor RNF43 have also been confirmed in three gastric cancer cell lines [57]. In addition, an association between loss of RNF43 and hyperproliferation has been shown. Accordingly, cancer cells with loss of RNF43 were more proliferative, leading to a higher Ki67 activity [57]. 

Epigenetic changes have also been reported to play a significant role in gastric cancer. For instance, APC is not only often mutated but is also commonly hypermethylated. Accordingly, methylation of APC occurs in 37.7% of healthy tissues but in 52.9% of gastric cancer samples [58]. The frequency of methylation of the Wnt antagonist Dkk3 has been reported to be enhanced by 20% [59] to 30% [60] in cancerous lesions. Furthermore, the gene coding for secreted frizzled related protein 1 (SFRP1) was hypermethylated in 44% of primary gastric cancer samples [61]. Additionally, hypermethylation of this gene was positively correlated to the loss of SFRP1 expression [61]. 

Another mechanism prominent in gastric cancer is the pathologic activation or inactivation of Wnt target genes via microRNAs [62]. A review summarizing 352 microRNAs found in gastric cancer reported that 41 of them were shown to be upregulated in at least two studies and 28 microRNAs were shown to be downregulated [62]. Among the most frequently reported downregulated microRNAs was microRNA-103, which regulates Axin2 [62,64]. Furthermore, microRNA-135, which regulates APC, has been reported to be upregulated by 1.6-fold in gastric cancers [62,63]. 

Studies also revealed the existence of single nucleotide polymorphisms (SNPs) in the CTNNB1 gene [65] as well as in the Axin1 gene [56], indicating that Wnt signaling may be related to the different congenital risks for developing gastric cancer. Interestingly, four of the reported SNPs in the CTNNB1 gene are associated with a higher cancer risk and only one with a reduced risk [65].

Among the investigations of Wnt signaling in gastric cancer, several studies have also focused on its role in the development of premalignant lesions. For instance, APC mutations were found already in low-grade dysplasia and were less frequent during the progression of gastric cancer [54]. These findings have been confirmed in mouse models, where deletion of APC or GSK3beta leads to benign lesions, such as polyps and adenomas, but not to malign lesions [14,66]. In addition, RNF43 mutations occurred in 35.2% of early gastric cancer adenomas [54]. Thus, it has been proposed that downregulation of RNF43 occurs rather early during carcinogenesis and that it is important for the transition from adenoma to carcinoma [54]. Furthermore, more than 80% of gastric cancer samples showed loss of heterozygosity at the RNF43 locus, which is an indicator for the loss of tumor suppressor genes [54].

Further experimental mouse work showed that upon *H. pylori* infection, mice with RNF43 mutations presented higher levels of gastric gland atrophy, hyperplasia and the gastric stem cell marker CD44 [67]. Consequently, mutations in the RNF43 gene increase the susceptibility of mice for severe *H. pylori* induced gastritis.

Additionally, histological studies that examined the nuclear staining of Wnt pathway components found nuclear staining for beta-catenin in one-third of gastric tumors [44]. As this staining was present in gastric cancer of the diffuse as well as of the intestinal-type, the authors also concluded that hyperactivation of beta-catenin is a common starting point early in the sequence of carcinogenesis rather than a determinant for the histological type of the cancer tissue [44]. Lgr5 expression has been explored in gastric lesions, and the strongest staining for Lgr5 was detected not only at the base of the glands but also at the luminal side of the tissue, where under homeostatic conditions only differentiated cells reside [68], and furthermore at the invasion front of the tumor [69]. This has been interpreted as an expansion and mobilization of the stem cell niche [68], pointing towards a critical role Wnt signaling in the context of invasion.

Experimental models have been developed to study the impact of aberrant Wnt signaling on the development of gastric pathology: K19-Wnt1 mice overexpressing Wnt1 in gastric epithelial cells developed “small preneoplastic lesions consisting of undifferentiated epithelial cells” in the stomach after 7 weeks and furthermore, the number of those lesions increased over the time [70]. 

Another group performed subcutaneous injection of AGS-Wnt1 cells in nude mice, which then developed significantly larger tumors compared to mice injected with AGS cells alone [46].

In a different study, upregulation of the Wnt signaling pathway was achieved via three different mechanisms, either by deletion of APC or GSK3, which are Wnt signaling inhibitors, or by inhibition of beta-catenin degradation in mice and they consistently found that small antral microadenomas appeared already 4 days post induction, which then enlarged over the course of time and overexpressed Lgr5 and Axin2 [14]. Additionally, the authors reported a loss of parietal cells as well as formation of “fundic gland polyposis interspersed with adenomatous change” which also showed increased nuclear beta-catenin staining [14].

In addition to its procarcinogenic effect, beneficial effects of inhibition of Wnt signaling in already established gastric tumors have been demonstrated in a xenograft model where injection of a Wnt5a-inhibitor into mice reduced the amount of liver metastases compared to mice that did not receive the inhibitor treatment [71]. Similarly, gp130^F/F^ mice that develop intestinal-type gastric cancer were treated with a Fzd inhibitor leading to an inhibited tumor growth [72]. 

## 6. Aberrant Wnt Signaling and Its Implications for Prognosis

The expression of Wnt target genes has been shown to correlate with the prognosis of gastric cancer patients. Wnt5A expression in gastric cancer patients is associated with more advanced stages of the tumor and a poor prognosis for the patient [49].

A meta-analysis performed by Huang et al. revealed furthermore that Lgr5 overexpression is significantly correlated to the T-stage of the TNM classification, as well as to the N-stage [73]. They found that patients with cancer lesions overexpressing Lgr5 had a significantly higher risk of mortality [73]. Also, within the group of patients with lymph node metastasis (N1), those with Lgr5+ lesions had a significantly lower 5-year-survival rate than those with Lgr5− lesions (54.4% vs. 89.4%) [74]. Moreover, gastric cancer patients with Lgr5+ tumors but without metastases at surgery were found to have a higher rate of recurrence or metastasis compared to patients with Lgr5− gastric tumors [69].

Furthermore, hypermethylation of Dkk3 has been found to be associated with higher mortality [60]. Additionally, the expression level of RNF43 was significantly correlated with the stage of tumor: Low RNF43 expression was associated with a low histological differentiation, bigger tumor size, deeper invasion and advanced pTNM stage [57]. Gastric cancers with low expression levels of RNF43 resulted in the worst prognosis for the cancer patient [57]. Apart from the advanced tumor stage that is associated with low RNF43 expression, RNF43 normally inhibits chemotherapy resistance in vitro, and this protective mechanism is eliminated by the loss of RNF43 [25]. Interestingly, the protecting effect of RNF43 by preventing the self-renewability of gastric cancer stem-like cells, could be partly reversed by adding R-spondin1 and Wnt5A in vitro [25], further supporting the concept of Wnt pathway overexpression leading to cancer initiation and progression.

## 7. Relevance of Wnt Signaling for Cancer Therapy

Due to its involvement in gastric carcinogenesis, modulating the (aberrant) Wnt signaling pathway could be a suitable therapeutic target, and several compounds are under investigation.

For example, DKN-01, a monoclonal antibody against the Wnt signaling antagonist Dkk1 is currently in phase 1 of a clinical trial for patients with gastric adenocarcinoma [75]. Other monoclonal antibodies that have been developed bind different types of the frizzled receptor [76] and some of them are currently investigated in phase 1 studies [77]. Furthermore, a polyclonal Wnt5A antibody has been reported to inhibit the Wnt-dependent internalization of receptors [71].

Another promising drug is IWP, a small molecule that targets Porcupine [78], an endoplasmic reticulum transmembrane protein that limits the secretion of Wnt [79]. Consequently, IWP might block the secretion of Wnt in tumors and thereby inhibit further tumor growth. At present, IWP is used in experimental settings and is also undergoing clinical trials [79,80]. 

Furthermore, colorectal cancer research revealed that a combination of peptide vaccines and anti-cancer drugs induced upregulation of the Wnt-inhibitor RNF43 [81], and this therapeutic scheme has been proposed for use in patients with gastric carcinomas, too [57].

While new strategies to target Wnt signaling in gastric cancer are emerging, so far there is no Wnt pathway targeting drug that has successfully passed all phases of clinical trials and is implemented as a therapeutic agent. This is probably because Wnt signaling is crucial for many physiological functions of the organism. However, new approaches, as outlined above, take advantage of specific alterations found in cancer tissue and are more likely to succeed. Alterations of Wnt signaling in patients with gastric cancer are heterogenous, and therefore, it will be important to apply diagnostic tools to identify the individual patient mutations to be then able to apply personalized strategy to target aberrant Wnt signaling. 

## 8. Conclusions and Further Perspectives

Wnt signaling has been shown to be a key mechanism for maintaining homeostasis of the gastric gland. Wnt signaling is essential for stem cell identity, epithelial turnover, and is a determinant of cell fate and cellular diversification within the gland. R-spondin3 is a critical regulator of Wnt signaling in the stomach, and its anatomically restricted expression in the gland base enables the maintenance of the Wnt gradient in the gland. This system allows a rapid adaptation of the tissue proliferation and its function, as has been demonstrated in the context of *H. pylori* infection. While this adaptation is beneficial for epithelial regeneration and injury repair, a deregulated Wnt signaling is a critical driver of gastric carcinogenesis. Alterations in the Wnt signaling pathway not only initiate cancer progression but can also be determinants for the prognosis of cancer patients. Therefore, Wnt signaling remains a promising therapeutic target in gastric cancer, with many new compounds that are being developed and currently investigated in clinical trials. 

## Figures and Tables

**Table 1:** Overview of Wnt pathway components dysregulated in the context of gastric cancer (GC).

**Upregulated Wnt Pathway Promoting Genes**		
Wnt1	Enhanced staining pattern in 98/180 of GC samples	[45]
	normal gastric mucosa < precancerous lesion < early gastric adenocarcinoma < advanced gastric adenocarcinoma	[46]
Wnt2B	In 2/8 GC samples	[47]
Wnt5A	Upregulated in 30% of GC	[48,49]
Wnt6	WNT6 expression associated with tumor stage and nodal status	[50]
Wnt10A	In 3/6 GC samples	[51]
beta-catenin	Upregulated in GC compared to tumor-free tissue (p = 0.0046)	[52]
**Loss of Function Mutations in Wnt Pathway Inhibitors**		
APC	In 7% of GC	[12]
	In 15–18% of GC	[53,54]
	In 30–34% of GC	[52,55]
Axin 1, Axin2	4/70 GC	[56]
RNF43	42/93 GC	[25]
	In 33% of hypermutated GC	[12]
	In gastric cancer cell lines	[57]
	in 35.2% of early gastric cancer adenomas	[54]
**Epigenetic Modifications**		
APC	37.7% in healthy tissues vs. 52.9% in GC	[58]
Dkk3	20/94 GC	[59]
	117/173 GC	[60]
SFRP1	44% of GC	[61]
**Regulation via microRNA**		
Upregulation	41/352 microRNAs: miRNA-135 (APC)	[62,63]
Downregulated	28/352 microRNAs: miRNA-103 (Axin2)	[62,64]
**Single Nucleotide Polymorphisms (SNPs)**		
CTNNB1		[65]
Axin1	5 SNPs in 70 GC samples	[56]

Adenomatous Polyposis Coli (APC), ring finger 43 (RNF43), Dickkopf 3 (Dkk3), Secreted Frizzled Related Protein 1 (SFRP1), Catenin Beta 1 (CTNNB1).
